# “Everything is revolved around me being heavy … it’s always, always spoken about.” Qualitative experiences of weight management during pregnancy in women with a BMI of 40kg/m^2^ or above

**DOI:** 10.1371/journal.pone.0270470

**Published:** 2022-06-24

**Authors:** Frankie J. Fair, Helen Watson, Katie Marvin-Dowle, Rachael Spencer, Hora Soltani

**Affiliations:** College of Health, Wellbeing and Life Sciences, Sheffield Hallam University, Sheffield, United Kingdom; University of Mississippi Medical Center, UNITED STATES

## Abstract

**Introduction:**

Maternal weight management services have been recognised as a good opportunity to influence lifestyle and dietary behaviour of mothers and families. Exploring women’s views of maternal weight management services is paramount to understand what constitutes the most suitable service. This study therefore explored experiences among women with a raised body mass index (BMI) of maternal weight management service provision and the barriers and facilitators to weight management during pregnancy.

**Method:**

Thirteen women with a BMI≥40kg/m² undertook semi-structured interviews around weight management experiences during pregnancy. Interviews were audio recorded and transcribed verbatim. Inductive thematic analysis was undertaken.

**Results:**

Four themes emerged. 1). "Understanding where I am at" showed current readiness and motivation of women varied, from being avoidant to being motivated to make changes. 2). "Getting information" revealed inconsistent information provision during pregnancy. Women particularly wanted practical advice. Some attempted to find this for themselves from friends or the internet, however this left some women feeling confused when different sources provided inconsistent advice. 3). "Difficulties I face" identified physical, emotional and financial barriers and the strategies some women used to overcome these. 4). "Encountering professionals–a mixed experience" demonstrated women wanted to be treated with respect and sensitivity and that how weight management information was addressed was more important than who provided it. The fine line professionals tread was demonstrated by women thinking that they had received inadequate information and yet too much focus was placed on their weight and the associated risks during pregnancy without practical solutions to their weight management challenges.

**Discussion:**

Women were empowered when practical advice was provided, not just the continual repetition of the risks of being obese during pregnancy. Antenatal weight management services need to be clear, sensitive and respectful. Services centred on individual women’s needs and on their current and previous experiences are required. The psychological and social contexts of weight management also need to be addressed.

## Introduction

Globally obesity and overweight (body mass index (BMI)≥25kg/m²) has been estimated to affect 38% of women [1], with rates varying by country. One recent study has found the proportion of women with the most severe form of obesity (BMI≥40kg/m²) to vary from 1.6% in Spain to 9.7% in the United States of America (USA) [2]. Alongside increased overweight and obesity in the general population over recent decades, maternal obesity during pregnancy has significantly increased [3,4]. Within England a recent cohort has shown 1.6% of pregnant women to have a BMI≥40kg/m² [5]. Furthermore, childbearing itself has been acknowledged to contribute to the rise of overweight and obesity in women [6].

Obesity during pregnancy has been associated with a wide range of adverse outcomes for both the mother and the infant [7,8]. For the mother this has included increased risk of pre-eclampsia [9,10], gestational diabetes [8,9], preterm birth [8], induction of labour [7], Caesarean Section [11] and surgical site infection [7]. The adverse outcomes noted for infants born to women with obesity during pregnancy include increased risk of being large for gestational age [8], admission to neonatal intensive care [7], stillbirth [11,12], neonatal mortality [11,13] and childhood obesity [14]. For all of these adverse outcomes women with a BMI≥40kg/m² were at the greatest risk; with their risk not just being higher when compared to women of normal BMI but also when compared to women with lower levels of obesity (BMI 30–39.9kg/m²) [15]. Overall, it has been estimated that 23.9% of pregnancy complications are attributable to maternal overweight or obesity prior to pregnancy [8].

Women with obesity prior to pregnancy have also been shown to be more likely to gain excessive weight during pregnancy [16], with a recent meta-analysis of individual participant data suggesting 44% of women with obesity gain excessive gestational weight [17]. Increased gestational weight gain has itself been associated with adverse maternal and neonatal outcomes both in the short and long term [18], including increased risk of Caesarean Section [17], induction [19], large for gestational age [18], poorer breastfeeding outcomes [20] and childhood obesity [14], especially when excessive weight gain occurred in women with obesity.

Traditionally pregnancy has been viewed as a good time to influence maternal healthy lifestyle as women are believed to be particularly receptive to healthy eating and physical activity messages at this time [21]. Furthermore, it has been seen as an opportunity to influence the long-term health of the woman and her family if changes made during pregnancy were sustained [22]. Many national and international guidelines have therefore recommended counselling women regarding healthy eating and physical activity during pregnancy [23–25]. However, a recent meta-review of lifestyle interventions during pregnancy has only found a minimal decrease in gestational weight gain and no other clear benefits on other adverse pregnancy outcomes from lifestyle interventions during pregnancy for women with overweight or obesity [26].

As the proportion of women with a pre-pregnancy BMI≥40kg/m² has continually increased globally, understanding how these women approach weight management in pregnancy and their attitude towards maternal weight management services is important. The context of the study provided additional significance given the region has been shown to have one of the highest rates of maternal obesity in England [27]. Deprivation scores have also been found to be among the worst in England, with indicators such as life expectancy, proportion of children living in low-income families and employment all falling below the average for England [27]. Previous research has also suggested potential differences in the experiences of women with a BMI of 30-40kg/m² and those with higher BMIs [28], highlighting the necessity for additional research among women with higher BMIs. While quantitative methods such as surveys can reach a greater number of participants and allow generalizability of results, qualitative research is considered the most appropriate methodology for providing context through in-depth exploration of the topic from the participants’ perspective [29]. The aim of this research was therefore to explore the experiences of maternal healthy weight service provision in women with a BMI≥40kg/m², alongside the barriers and facilitators experienced by these women in weight management during pregnancy.

## Methods

### Theoretical framework

This study used a qualitative interpretive approach, from a constructivism philosophical position as the aim was to understand the meanings the women created and attributed to their experiences [30]. Ontologically the study was grounded in relativism, as the researchers acknowledged that multiple realities exist which are subjective and shaped through individual lived experiences. [30,31]. At an epistemological level, a transactional position was adopted. This recognized that the researchers did not come into the research process as blank slates but brought with them their own previous histories and perspectives of weight management [30]. It was recognised that this could impact on the interpretation the researchers formed. Therefore, to ensure trustworthiness of the research reflexivity was undertaken, where the researchers critically reflected on how their social background, assumptions, positioning and behaviour impact on the research process [32,33].

Within the social sciences multiple competing theories exist which allow a phenomenon to be viewed from multiple perspectives, with each perspective providing a reasonable explanation of a phenomenon [34]. This research was influenced by aspects of both the Capability, Opportunity, Motivation, Behaviour (COM-B) model [35] and the socio-ecological framework [36]. The COM-B model views behaviour to be generated from an interaction of capability, motivation and opportunity [35], while the socio-ecological framework views behaviour being influenced by multiple levels of factors including intra-personal, inter-personal, organisational, community and public policy factors [36]. These methodological and theoretical orientations were used to interpret and understand experiences among women with obesity of weight management services and their barriers and facilitators to weight management during pregnancy.

### Study setting

The exact name of the study setting is not included for data protection and anonymity purposes. The study was conducted in a region within Yorkshire and Humber with high rates of maternal obesity and deprivation compared to the rest of England [27].

### Recruitment and data collection

A purposive sampling strategy was used. Pregnant women who had a BMI≥40kg/m² when booking for antenatal care were approached at their 36 weeks gestation anaesthetic review appointment between December 2018 and February 2019. The 36 weeks gestation review appointment was chosen as that was the last universal appointment at the maternity unit for women with a BMI≥40kg/m². Women were offered an interview (face-to-face or over the telephone) or participation in a focus group. All women chose an individual interview. Interviews were semi-structured using an interview schedule that covered weight management advice given during this pregnancy, awareness of services and facilitators and barriers to weight management during pregnancy (see S1 File). The schedule was developed in collaboration with a maternity service user group to confirm acceptability and clarity to a wide audience. All interviews were audio recorded.

Nineteen women expressed an interest in participating in an interview. These women were followed up with either 2 emails or 2 telephone calls depending upon their stated preference. After this no further contact was attempted. In total 13 women completed an interview. All interviews were undertaken by a female interviewer. After 13 interviews no new concepts were emerging, therefore data saturation was felt to have been achieved and further recruitment stopped.

### Data analysis

Interview data was transcribed, anonymised and managed using NVivo. An inductive or ‘bottom up’ thematic analysis approach was undertaken without trying to fit the data onto a pre-defined coding structure or theoretical framework [37]. An inductive approach allowed the themes to be determined from the data rather than the researchers’ preconceptions [37]. A systematic methodology was used for identifying, analysing and reporting patterns within the data, applying the six-phase process described by Braun and Clarke [37]. After familiarisation with the data, two researchers independently open coded the transcripts line by line to summarise the elements discussed. These initial codes were close to and derived from the text. Both researchers individually grouped and refined the initial codes into categories. From these categories and through discussion the researchers agreed the themes and sub themes emerging from the data. All coded data was then fitted within these themes to ensure completeness of the analytical themes and inclusion of all relevant data. After further reviewing and revision of the generated themes, clear definitions and names were given to each of the emerging themes.

Development of the themes was iterative and included a reflexive process where the researchers acknowledged their pre-understanding and biases to ensure the themes remained close to the original data. The complexity of women’s experiences was reflected by describing within each theme the contradictory aspects and diverse nature of the multiple realities of the interviewees. Extensive direct quotations have been presented to illustrate and confirm the researchers’ interpretations within each theme and subtheme. All interviewees were given the option of commenting on the initial interpretation for member validation; with four women choosing this option. The initial themes to which their data had contributed were sent to these women to confirm their own viewpoint was represented within the researchers’ interpretation.

### Ethical considerations

Ethical approval (IRAS 17/EE/0378) and research governance approvals were obtained prior to commencing this study. Written informed consent was obtained from women prior to undertaking the interviews. Women were given a £10 voucher to compensate the time they had given to participate in the interview. Pseudonyms have been used to protect confidentiality.

## Results

Of the 13 women who participated, nine undertook face-to-face interviews and four telephone interviews. All women were in the late third trimester of pregnancy. Five women were primigravid, seven women had one previous child and one had 2 previous children. Three women had experienced care in a different hospital Trust during their previous pregnancy, while the other women had received care within the same hospital Trust for all of their pregnancies. Six women had other people present during the interview, three had their partner present, one their child, one their mother and one both their mother and their partner. However, these companions only contributed to the interview on three brief occasions and these comments were not coded within the analysis.

**Fig 1:**
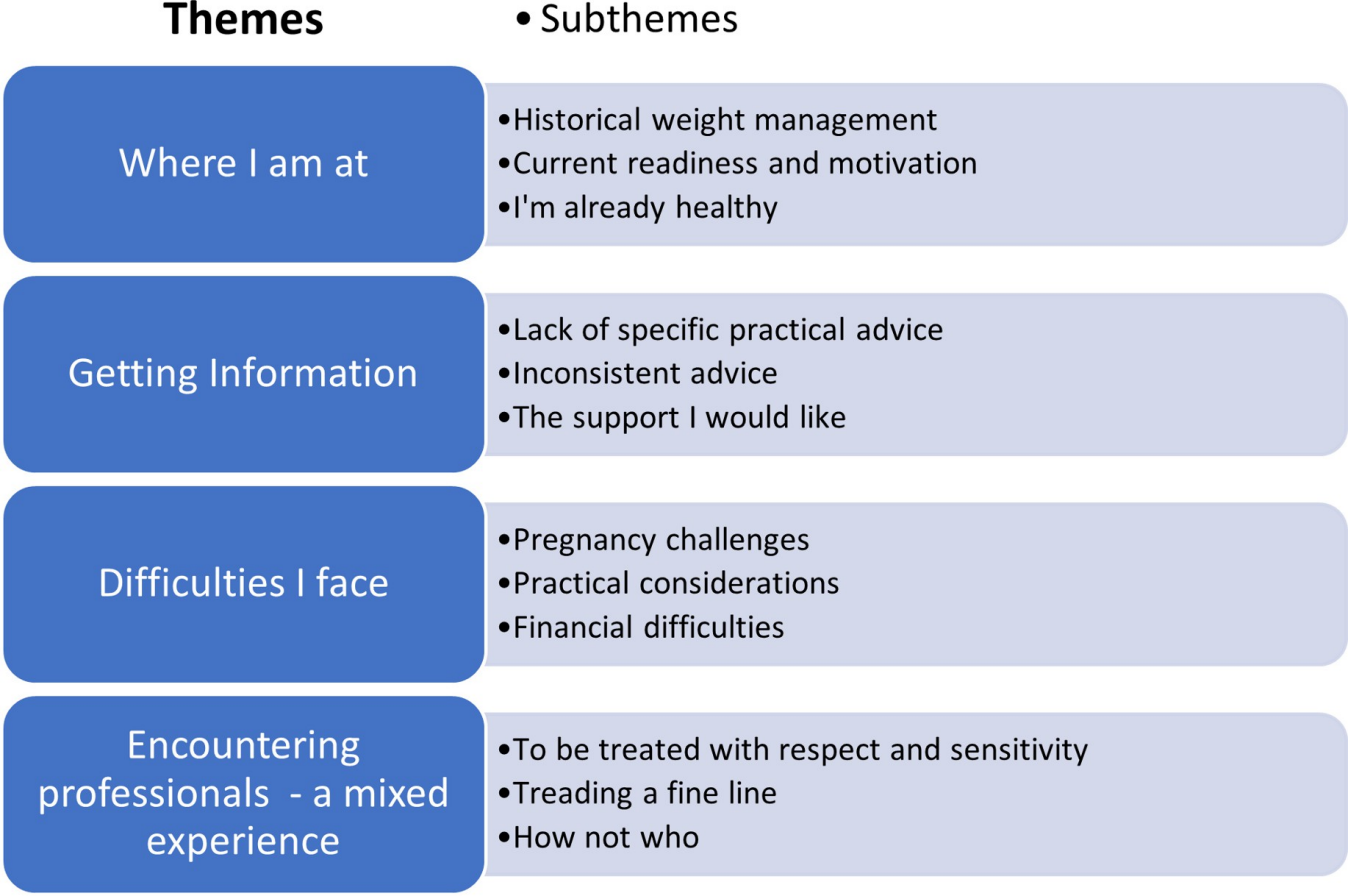
Themes and subthemes within the analysis.

Four themes emerged from the data; “Where I am at”, “Getting Information”, “Difficulties I face” and “Encountering professionals–a mixed experience”. Fig 1 illustrates the themes and subthemes.

### Where I am at

#### Historical weight management

Women were noted to be at different stages on their weight management journey, each bringing their own story of weight management over the years. Over half of the women described previous efforts at weight management with the majority using weight management support groups, seeing dieticians or participating in exercise such as going to the gym. Some had also used weight loss shakes, tablets, or followed special personalised diets. Most had seen success in their previous attempts at weight management although many had seen weight return with pregnancies or over time.

*“I used to do Slimming World before … I have tried like pills*, *I had stuff like that*. *I have been to dieticians and tried the gym*.*”* Danielle

#### Current readiness and motivation

Some women were already following their own weight management plans prior to and during pregnancy, noting the fit of certain clothes or frequently weighing themselves to monitor their own weight gain. These women were motivated to prevent excessive weight gain which would need losing again post pregnancy. The recognition that everything they were eating was being passed onto the baby also motivated some women to manage their weight during pregnancy.

*“I have been watching my own weight anyway because obviously … I put a lot of weight on in my first pregnancy and then I’ve not really got it off*, *so I don’t want to put any more on in this pregnancy*.*”* Joanne

*“It’s [my diet’s] changed during pregnancy because I’ve started eating more healthier … Making it better for the baby*.*”* Emma

While acknowledging a need to address their weight, some women did not feel ready to at present. These women talked positively about their plans to lose weight in the postnatal period by increasing exercise or starting to attend a commercial weight management group (private company offering weight management support that the women could independently access and finance). Other women however did not want the issues of weight management being raised, for example declining offers of support from dietetic services or not wanting to receive information from community midwives around healthy eating or physical exercise. Several women linked their avoidance of weight management to their low self-confidence.

*“I think most women who are overweight and pregnant just feel low in themselves* … *I don’t know about anyone else*, *but from my experience that’s how I feel*.*”* Rebecca

Whether or not women felt ready to receive weight management support, they all recognised that the motivation to manage weight had to be internal, with services only able to be effective if the woman herself was ready to address her weight.

*“You can tell somebody something*, *but if they don’t want to do it they will not do it*.*”* Danielle

For most women, their readiness to receive weight management advice appeared to influence their acceptability of being weighed during pregnancy. The majority were happy to be weighed at appointments and felt weighing should be a part of routine practice. In contrast a few women were ambivalent, not minding one way or the other and two women felt uncomfortable with being weighed for example feeling anxious about how much they would weigh and not wanting to know the figures.

*“I have seen a dietician as well with the diabetes side of things*. *It’s been really useful actually* … *I get weighed at every appointment that I go to … I’m happy to have that*.*” Zoe*

*“I honestly don’t mind [being weighed] because in my opinion it is what it is*, *I know within myself if I’ve been over-indulging with things*.*”* Laura

*“I don’t like being weighed*, *but they have got to do what they have got to do*.*”* Lindsay

#### ’I’m already healthy’

Numerous women described themselves as already healthy, eating healthily and exercising in the recommended way. They were happy with the way things were and did not see a need to address weight management. Some therefore felt professionals could offer them no further advice and found it difficult when professionals assumed that their obesity was due to current unhealthy eating.

*“Some people are quite happy being the way that they are*, *and they don’t necessarily feel like they need to change*.*”* Samantha

*“It was very much assumed you must be eating too much or you must be not moving enough*. *But actually*, *I’m more active in pregnancy and I’m eating healthier in pregnancy because I’m focusing on the baby*.*”* Natalie

It was however noted that the diets several women described as ‘eating healthily’ didn’t appear to fully fit current healthy eating recommendations:

*“Well jacket potatoes*, *tuna pasta*, *do you know what I mean*. *I don’t really like salad or vegetables you know what I mean just healthy food*.*”* Joanne

### Getting information

Women described a lack of information provision during their pregnancy, confusion when inconsistent information was given and the information they would have liked to receive.

#### Lack of specific practical advice

Information provision experiences differed between women. Many reported receiving no information regarding weight management during their current pregnancy. When probed further some of these had received information, however information provision was minimal for example just being handed a leaflet or being told verbally the foods they should or shouldn’t eat while being pregnant. This information was usually provided at the booking appointment alongside a plethora of other information and women therefore felt the information was ‘glossed’ over or it remained in a pile of unread leaflets. This had led to the impression that they had received nothing.

*“I have been given leaflets about weight*, *but I’ve just gone home and then I’ve put them on the side and just well obviously they’re still in the pile*.*”* Joanne

Those who had received information mainly received it from their community midwife, with two women with gestational diabetes also receiving information from a dietician and another couple of women at a hospital midwife appointment. Examples of information provided around healthy eating included the eat well plate; advice to eat fruit, vegetables, wholegrain foods or complex carbohydrates; advice around portion sizes; avoiding snacks and too much sugar; discussions around iron rich foods; not to try to lose weight during pregnancy and to eat ‘everything in moderation’. Very few women received information on exercise during pregnancy and where they did, it consisted of being told to walk, swim or use the treadmill at the gym.

*“I’ve also noticed that this time round there’s not been a lot of information on your group exercises*. *Like before they’d tell you* … *aqua aerobics or stuff like that and this time I’ve not really had that*. *So I think for women in general I think they need that information of what they can do*, *where they can go*.*”* Rebecca

Women wanted specific advice such as menu ideas, meal plans or exercise plans rather than having to try to work out for themselves what would be beneficial for their body and the baby. Women also wanted practical advice on weight gain in pregnancy, especially what target weight gain they should aim for and how to achieve it. Several women had found USA recommendations regarding gestational weight gain on the internet; but were unsure if those guidelines were the correct ones for them to follow.

*“A meal plan*, *like a weekly example … meal ideas and meal plans*, *so you know you can follow*.*”* Claire

*“For me the weight gain is important because I’m big anyway*, *so I want to know how much you’re going to gain or what’s going to make you gain*. *So you can then work on right well if that’s what I’m going to gain then I need to cut down and do more exercise*.*”* Rebecca

*“They don’t tell you what’s the average [weight gain]*, *like what’s a good weight gain*, *what’s a terrible weight gain*, *… what’s going to make things difficult*. *It was very much a right you are overweight already*, *there’s not much else we can do* … *It wasn’t like a how can we prevent you gaining too much weight that it then becomes difficult for you to do anything*?*”* Chloe

While women wanted practical information a few women felt that professionals could only really offer general advice. The guidance that pregnant women should not lose weight or go on restrictive diets, prevented specific support being provided.

*“I think sometimes when it comes from like a professional*, *there’s not really that much that they can offer*, *or that’s how it feels*, *because I don’t think much can be done to a certain extent but it’s just advice isn’t it*.*”* Alice

For many women the times they were weighed were seen as a missed opportunity to discuss progress and offer weight management advice, as all too often the professionals just noted down their weight with no discussion. Women appreciated being encouraged when their weight gain was minimal or being provided with practical examples of things they could do about excess weight gain such as eating foods that were more filling to reduce the requirement to snack. Instead, they often felt that professionals overlooked their success during pregnancy.

*“With my community midwife … she will tell you how much you have put on and whether that’s good*, *bad*, *maybe slow down*, *maybe you need to eat some more food*. *Whereas here they just sort of write it down and nobody discusses nothing*.*”* Chloe

*“I lost over a stone between Christmas and new year because I was poorly*. *No-one seemed to really acknowledge any of that*. *It was just really odd*.*”* Michelle.

*“My midwife*, *she’s been very encouraging in the fact that I haven’t put on much at all*, *she’s been really* …*you know encouraging me just to stick with whatever I’m doing*.*”* Natalie

For some the information they received during this pregnancy was deemed adequate because of the advice they had received during previous pregnancies. They therefore felt advice should be offered more intensively to first time mothers with optional sessions for subsequent pregnancies, as some felt it took a while to become familiar with the new concepts introduced.

*“If this was my first pregnancy and I didn’t go there [specialist clinic] I wouldn’t know*, *I wouldn’t know what foods would be good for me and the baby*. *I wouldn’t know what sort of exercises to do*, *because that’s another thing that she told me about*.*”* Rebecca

Inconsistent advice. Clear information that was consistent between professionals was received by some of the women. However, many experienced inconsistencies in information, for example healthcare providers telling women that the information received from other healthcare providers was incorrect or differences in the information found in online parenting forums among women from different geographical areas of the country. One woman even reported feeling that she had to explain to a professional the policy of referring women with a raised BMI to consultant led care. It was felt one consistent set of information would be helpful to avoid women feeling confused as to what was correct.

*“I think it’s just a case of consistency across everybody*. *So that everybody is … reading from the same book*. *I am getting information from one side and information from another side* … *and then they don’t talk to one another*. *So it’s [this] one information–[that] one lot information*, *that never even matches up*.*”* Chloe

Women also found it difficult to work out what to do when professionals told them to eat healthily but not to lose weight and to allow themselves treats. It left them uncertain of what actions they should actually take.

*“I think you’re between a rock and a hard place to be honest … They advise you not to diet but to eat healthy but to still treat yourself and you’re kind of like*, *what do I change*?*”* Alice

#### The support I would like

Electronic resources were particularly desired where women could access clear information at their own pace, rather than feel overwhelmed by information during appointments. Many women reported finding information for themselves online or through apps anyway, for example healthy alternatives to cravings and weight gain guidance, but often wished they had found such information earlier on in their pregnancy.

*“A website that they can go on and look because most of the time when you’re in your appointments everything just kind of goes in and then you forget certain points of things*.*”* Laura

Feeling socially isolated was also reported, with some women wishing for a group with other women who were larger like them.

*“I found that there’s not many big girls to talk to*, *that kind of thing*. *I always felt I was on my own*, *that there were a lot of smaller women around in the clinics and … it felt like you were a bit on your own*.*”* Claire

Women felt weekly group support where their progress could be monitored would be beneficial; particularly peer support groups where others could share their encouraging stories, rather than just professionals discussing information leaflets. Several women felt that their attendance at a commercial weight management group, which had adapted healthy eating plans for pregnancy, had been invaluable for getting advice and in providing support for sticking to a healthy diet. A desire was also expressed by some women to be informed about forums for women with similar pregnancies where they could offer each other support and advice.

*“If you’re in an environment where you’ve got support from other people who are going through it as well or*, *like*, *they share their success story*. *Rather than just maybe sitting at a desk and … having a midwife*, *like*, *just hand you a leaflet showing you what you should be eating and what you shouldn’t be eating*.*”* Samantha

A few women however voiced that they would not have the confidence to attend group sessions where their weight would be discussed in front of others.

*“Some people aren’t confident being around other people with their weight issues as well*, *like me*.*”* Emma

### Difficulties I face

#### Pregnancy challenges

Cravings during pregnancy were a struggle for many women as it was recognised that they craved unhealthy foods. Women wanted more advice on how to control these and appreciated times practical guidance had been given on healthy ways to obtain the nutrients craved. One woman had sought this information for herself, finding out what vitamins or minerals the craving may indicate she was lacking and finding healthy alternatives to meet this need.

*“I’ve got a huge thing for pork*, *so I’m just constantly eating*, *bacon and sausages and pork chops and ham and that’s my main protein source I think at the minute* …. *I’ve always quite enjoyed bacon*, *but not as much as literally every day I have to have a bacon bagel*.*”* Natalie

*“This time I’ve not craved sweets and chocolate and stuff like that*, *it’s been the opposite really … It’s made it loads easier*.*”* Joanne

The common ’eating for two’ myth was endorsed by many women’s peers or families. While many knew it was not true, others wanted more concrete information and explanation to help them to follow correct advice.

*“You hear it from people*, *oh it’s okay you can have that extra cake because you’re ‘eating for two’*.*”* Laura

*“I suppose that they do always try and say*, *you know*, *like*, *try and reduce your BMI*, *eating healthy*, *don’t do all this ‘eating for two’ kind of things*, *which I definitely did in my first pregnancy because sometimes I think you might—you’re just a bit oblivious to it or a bit naïve towards the information that they’re giving you and the reasons why they give it you*.*”* Samantha

Other women found it emotionally difficult to address weight management while pregnant as they knew they would gain weight as their baby grew. Others described finding it just too difficult to think about a strict diet while pregnant. Furthermore, women noted weight management was difficult due to excessive tiredness in early pregnancy, needing to eat during the night due to hunger and difficulties in exercising when advanced in pregnancy.

*“It’s hard isn’t it in pregnancy especially when you get further on*, *I think you get a bit lazier don’t you and you get bigger and you’re not exercising*, *you’re sitting a lot*.*”* Joanne

*“I say it’s [watching weight’s] one of the last things I wanted to really focus on*, *having to like be strict with diets*, *especially with having a toddler as well*.*”* Laura

#### Practical considerations

Many women were unaware of community-based services that they could access while pregnant to support them with healthy lifestyle and weight management. Furthermore, one woman who was aware of such groups reported barriers to accessing them as she was told pregnant women could only access the group if they were already members prior to pregnancy.

Women also voiced difficulties with eating healthily due to personal taste and difficulty in accessing fresh rather than convenience food, especially in deprived areas. Time pressures with a young family or when trying to work were also noted to restrict their ability to access social support groups, exercise activities or healthy eating. However, those with young children noted they were more active than in previous pregnancies as they had an active child to follow around or needed to be physically active to undertake the school run.

*“I do confess I am not a healthy eater because I don’t like veg*.*”* Lindsay

*“Now with the school run and I’ve got my little one who’s not in his pushchair no more*, *he wants to go for walks so now I’ve got the extra time I’m just out and about more*.*”* Rebecca

#### Financial difficulties

Financial constraints were also discussed, particularly the cost of buying healthy foods especially if they had to consider their whole family and not just themselves.

*“It’s hard being healthier anyway because I mean if you’ve*, *if you have a family*, *fruits not cheap …if you have got to eat seven a day or five a day now like*, *but it’s not cheap to do that*.*”* Lindsay

Women also voiced that finance restricted their attendance at commercial weight management groups, especially given the emotional difficulty of paying out money to see themselves gain weight. More co-operation between hospital Trusts and community commercial groups was desired for example offering free sessions to women during pregnancy. Impending maternity leave made financial matters even more pertinent to some women.

*“I know that I did stop going to Slimming World to pay to weigh because for me it was disheartening paying £5 every week when I know that it’s going to say that I’m gaining weight*.*”* Samantha

### Encountering professionals–a mixed experience

Women wanted to be treated with sensitivity when discussing weight management. However, there was a fine line for professionals with women describing both too much and too little focus on the issue. In the future, how advice is provided was considered more important to women than by whom.

#### To be treated with respect and sensitivity

The way some professionals talked to women made them feel uncomfortable, patronised and stigmatised due to their weight. Being spoken to in a demeaning or judgemental way made women feel worse. In contrast a professional’s positive attitude was appreciated by women and enabled them to think about making changes.

*“I just find it quite judgemental here*. *Very judgemental here … Everything is revolved around me being heavy*. *And it’s always*, *always spoken about*. *That it’s kind of like well this isn’t a new risk factor*!*”* Michelle

*“I know obviously when people are overweight or whatever it carries a stigma with it*, *but I think the way that people speak to you and the way it comes across*, *it should be looked at more*, *if you know what I mean*, *because if someone’s awful to you and they say it in a negative way you’re going to leave … feeling like crap*. *Whereas if someone talks to you really nicely and just says look you just need to do this or whatever then you don’t really like worry about it as much*.*”* Alice

All too often women also felt their concerns were dismissed and they were not listened to. They felt policy was frequently adhered to in a rigid way without listening to or addressing women’s concerns or viewing the woman as an individual. Women also reported questions they had around the risks of being obese while pregnant or how to mitigate these risks were left without clarifications.

*“I don’t seem to get listened to about that [questioning a policy] which is really frustrating*.*”* Michelle

Professionals were appreciated when they were friendly, interested, sensitive, encouraging and had time to listen, explained things well, answered women’s questions or concerns and provided non-judgemental information. Good communication between the woman and the healthcare professional and between different healthcare professionals helped women to feel supported. Health professionals were especially valued where they were committed to help the woman achieve a good result, for example through positive reinforcement of the healthy changes the women had made or wanted to make.

*“I sat and spoke to the Dietician*, *she gave us pointers of what kind of food to avoid and how much to eat of it which*, *she was really quite friendly and helpful*.*”* Claire

*“I didn’t feel uncomfortable*, *it was really relaxed and it was just nice to talk to someone that wasn’t patronising or looking down at you*.*”* Rebecca

#### Treading a fine line

Women reported simultaneously not receiving enough information around their weight, but also that too much focus was placed on it. Women described excessive focus on the risks associated with their raised BMI during pregnancy along with their need for consultant led care during pregnancy. These risks were often discussed at every appointment, leaving them upset and worried. Moreover, women felt frustrated when these risks were highlighted without any practical advice regarding eating, exercise or weight management. Women also reported feeling that healthcare providers assumed that they would have all of the complications associated with a raised BMI.

*“It’s consideration of how that person might be feeling*. *I was already quite nervous and when you are coming all the time hearing like risk*, *risk*, *risk*, *it doesn’t help*. *It really doesn’t*.*”* Michelle

*“When I was going [to appointments] I was like right here we go again and you knew what they were going to say before they said it*, *but at the same time they didn’t really give you any information in regard to changing anything … it was more kind of … that I was overweight not the fact that you needed to do anything or anything like that*.*”* Alice

*“I find it quite amusing when I go for my appointments and it’s always the same midwife that sees me*, *there’s always an element of surprise when they take my blood pressure*, *oh*, *oh it’s perfect*, *it’s very good*. *It’s like they expect because I’m bigger that my blood pressure is going to be too high*.*”* Natalie

After having a scan suggesting the baby was putting on weight rapidly one woman reported:

*“They were like you have got to go*, *you have got diabetes because you are so big*, *you have got to go and get this checked*, *and I haven’t*. *But that’s how it was attributed*, *that there must be something physically wrong with me because I am big … I just felt really bad when I left*, *… it kind of makes you feel quite deflated*. *… Quite anxiety raising*.*”* Michelle

This constant raising of weight as an issue meant some women felt like not attending further antenatal appointments. This was especially reported when women saw different people at every hospital appointment, with each new individual repeating the same information around the risks associated with a high BMI.

*“I think in the first pregnancy they were a lot*, *kind of*, *more forceful with it*, *in letting you know … Every appointment it was brought up and it really kind of upset me the first time … to a point where I was saying to my partner they should have*, *like*, *something on your chart that says you’ve discussed it*. *You don’t need to hear it every time you go because you already know*. *You’re aware that you’re overweight*, *you don’t need somebody to tell you at every appointment*, *which they did*.*”* Alice

In contrast women reported that some professionals wanted to avoid the issue, either not wanting to talk about weight or assuming the woman didn’t require support. This was especially difficult when women themselves reported finding it difficult to ask for help, so appreciated professionals proactively addressing weight management and making them aware of different options available. One lady who received more intensive support in a previous pregnancy really missed this additional advice in her current pregnancy.

*“I found xxxx [current] hospital to be a lot more helpful towards me*, *whereas xxxx [previous hospital] just left me to it*.*”* Zoe

*“I’ve missed the advice and the encouragement because* … *in this one [pregnancy] I’ve not really seen a lot of people this time*, *it’s like they’ve just let me get on with it … I was quite a bit* … *gutted I guess that I wasn’t transferred there [maternal obesity service]*.*”* Rebecca

Many women felt the focus of maternity care appointments was on the health of the baby. They longed for more focus on them, to reassure them that they were gaining the right amount of weight and to provide advice so they could create the best environment for their baby.

*“Nobody seems to really give a toss that I’ve been so poorly … They just kept saying well the babies still gaining weight*, *so you are okay*, *that’s all they kept saying*.*”* Michelle

*“I think it’s got to be a 50/50 where you’re being looked after as well as your baby*. *We know your baby’s fine*, *we know that*, *but what about the mum*?*”* Rebecca

#### How not who

Given the difficulties they had previously encountered, in the future it mattered more to women how weight management was approached than who provided advice. Women desired weight management advice from someone with whom they could establish a relationship and who could follow their progress. For many women the ideal person to do this was their community midwife whom they developed a bond with throughout pregnancy. Women felt that community midwives knew them as an individual and listened to their concerns. For others they would also be happy to have weight management discussed by dieticians or health visitors or other professionals such as commercial weight management group consultants. People who had achieved effective weight management themselves were considered to be the best people to offer advice.

*“The midwife actually I find that I have a good bond with her*, *I trust her opinion*. *And if she were to suggest something to me*, *I would take it on board*.*”* Zoe

*“I think I just feel like maybe the Slimming World consultants*, *especially if they’ve experienced it themselves*, *they’re the best people to give you the advice*.*”* Samantha

## Discussion

This study highlighted women with a BMI≥40kg/m² felt there was too much emphasis placed on the potential risks that they faced during pregnancy, which left many feeling anxious and stigmatised by healthcare providers. Women also reported receiving inconsistent information with insufficient provision of clear practical advice. Furthermore, women were all at different stages of readiness to address weight management during their pregnancy.

Within this study women reported an overemphasis on the risks associated with being obese during pregnancy. Current United Kingdom (UK) National Institute for Health and Care Excellence (NICE) guidance [24] recommends women with a BMI≥30kg/m² should be advised of the risks of her being obese during pregnancy for both the health of the mother and her infant. However, it appeared that this guidance led some women within this and previous studies to describe that their weight and the associated risks were focussed on repetitively [38,39] rather than receiving clear information on what to do about it. Others have particularly noted an emphasis on the risks for women with a BMI≥40kg/m² [38]. Furthermore, women described that because they were at increased risk it was assumed that they would experience all of the associated complications [40–42].

Health services’ focus on risk management, rather than taking individual factors into account, coincides with an increasingly prevalent medical model of care for these women. This model views pregnant women with obesity as needing to be ‘managed’ by obstetricians [43,44], as professionals becoming progressively interventionist in an attempt to protect themselves from litigation should an adverse outcome occur [45]. However, this focus on risk is at odds with women’s own focus, as clinical outcomes only cover a subset of the factors they consider to be important during pregnancy [46]. A systematic review of patient reported outcomes has shown that women with obesity viewed adequate healthcare provider support and an emphasis on their emotional wellbeing as key elements of care [46]. A move towards a social model of maternity care is argued for which integrates women’s physiological, psychological and spiritual wellbeing; with women and professionals working in partnership to support women to focus on health promoting activities [44]. The social model views the three most significant factors for women during pregnancy and childbirth to be choice, continuity of care and control [47], with maternal satisfaction an important outcome of pregnancy, not just a live, healthy mother and infant [48].

Treating women with a raised BMI as ‘high risk’ and in need of additional monitoring or prevented from accessing certain options such as having a waterbirth can increase their feelings of stigmatisation [41–43,49]. Stigma itself has been associated with poorer maternal health behaviours, mental health and stress, all of which have a negative impact on infant outcomes [50]. The stigma women face has also been identified as an issue within almost all of the previous studies and reviews read [28,38,40,49,51–58]. Stigma is particularly perceived if women feel weight management advice is offered solely due to their size not due to their need [43] or when professionals assume that obesity is due to a current lack of exercise or poor eating habits [39,40,53,55]. Despite evidence showing that BMI does not provide a full picture as it cannot differentiate adipose tissue from lean body mass [59], health professionals still place an over emphasis on BMI alone. Furthermore, it has been shown that it is possible to be ‘fit and fat’, with fitness being more important than fatness for long term prognosis [60]. Of note however is that women felt equally dissatisfied with the care they received when healthcare professionals avoided the topic of weight. Others too have noted this to be an issue due to professionals’ worry about women’s potential sensitivity and due to their own discomfort about addressing weight [28,39,51,52,56,61,62]. A lack of change in the concerns that women have highlighted regarding stigmatisation over the last decade emphasises how essential it is for enhanced healthcare providers’ training, to raise their confidence to discuss weight with women and to approach weight in a way that avoids stigmatisation.

This study has highlighted the lack of uniformity in the care that women with a BMI≥40kg/m² received around weight management during pregnancy, even within one hospital Trust over a short period of time. While a few received good information around healthy eating, physical activity and weight management during pregnancy, the majority of women described inadequate information provision. The lack of consistency in advice may in part be because of a lack of clear national guidance, as well as due to limited resources. The emphasis on risk without concurrent provision of practical advice noted in this and other studies, left many women feeling dissatisfied, disempowered and feeling guilty about their weight and the implications this may have on the outcomes of their pregnancy [49,51,55,62]. Women’s information needs around weight management during pregnancy not being met by healthcare professionals has been a recurrent theme within the literature among women with obesity [28,38,52,54,56,61]. A lack of information around gestational weight gain has been particularly evident [39,51,52,54] especially in areas without a bespoke weight management service [38].

The information many women described wanting was clear, practical, consistent advice that incorporated clear strategies for them to implement, not just telling them of the things they shouldn’t do. Others too have found women with obesity to want constructive advice, for example around the contents of a balanced diet [62], specific nutritional components required during pregnancy [53] or how to manage common pregnancy conditions such as morning sickness and cravings [51]. In contrast both in this study and others, some women reported feeling that they received no new information from professionals around weight management [43,55,62]. While the focus of professionals was on adhering to policy and advising women of the risks associated with being obese during pregnancy, women’s focus was more on their individual needs and on their requirement for practical advice. As a result, women frequently perceived that their informational and support needs were inadequately met by professionals. Provision of information tailored to the individual woman’s needs is therefore essential.

Many of the women who had previously had a baby voiced regret for the choices they had made in their earlier pregnancies, only realising in retrospect that it was inadvisable to ‘eat for two’ during pregnancy and that the excess weight gained in pregnancy would need to be lost after the birth. This is supported by evidence that primigravid women with a BMI≥40kg/m² gain significantly more weight than multigravida women [17]. Many women have also been noted to almost double their calorie intake during pregnancy [63]. The difference in information needs between first and subsequent pregnancies was recognised by several women. One parous woman was very grateful that she had received support in her first pregnancy even though she had not received the same support during her current pregnancy and others raised the potential requirement for more intensive information provision during the first pregnancy.

A further theme identified within this current study was ‘where I am at’ depicting the need to respect women’s previous weight management attempts and the influence they may have on her readiness to accept or enact advice provided during her pregnancy. The continual focus for women outside of pregnancy had been around losing weight, the change in advice to not losing weight during pregnancy but minimising weight gain left some women uncertain of what they could or should be doing. Others too have recognised that women often have a long history of trying to manage their weight [38,39,52,53,55]. The majority of the studies we reviewed that reported this aspect were noted to have included either exclusively or a high proportion of women with a BMI≥40kg/m². Of interest was that some women, both in this study and in previous research, defined themselves as healthy. For example, women have described following healthy eating advice such as eating fruit and vegetables or running 10 km; emphasising these healthy aspects as countering the risks of obesity during pregnancy [42,43,51,55]. This further highlighted the need for individualised care and sensitivity when providing advice, without automatically assuming that current energy imbalance is the cause of the woman’s obesity. Furthermore, several women within this and previous studies have described being unable to think about their weight during pregnancy due to feeling that weight gain was inevitable, but they had plans to address weight management in the postnatal period [52,54,55,58]. A lack of focus by healthcare providers on the postnatal period has however been shown, with pregnancy weight management support ceasing once the baby is born [38,55]. Assisting women to address weight management in the postnatal period could help to reduce their BMI prior to any subsequent pregnancies. This is essential given that pre-pregnancy BMI has been shown to be a stronger predictor of adverse outcome that gestational weight gain [64].

Historically pregnancy has been viewed as a ‘teachable moment’ for women due to changes mainly in their motivation, related to their concern for the developing fetus’s health [21]. Women’s frequent contact with health professionals during pregnancy also provides an opportune time to deliver health promotion [21]. The COM-B model however sees behaviour as having three determinants not just from motivation, but also capability and opportunity [35]. Furthermore, the socio-ecological model views multiple factors influencing behaviour including the personal, organisational, community and public policy [36]. This research clearly showed that while some women were motivated during pregnancy to change their lifestyle, not all women were personally motivated simply because they were pregnant. Women’s motivation could also be impacted at an organisational level, if they felt stigmatised or unvalidated by healthcare providers. Women’s psychological capability to achieve weight management during pregnancy was decreased due to inadequate information provision, in part because of healthcare providers lack of knowledge and skills leading to their avoidance of conversations or the provision of inconsistent information at the organisation level. Barriers were also noted to the women’s physical capability due to personal factors such as the associated tiredness and cravings in pregnancy, but also due to inter-personal factors such as lack of childcare. Women’s physical opportunity to achieve weight management during pregnancy was impacted personally by financial and time constraints, but also at the community level due to a lack of access to healthy foods or suitable support groups. This study also emphasised the influence on women’s social opportunity to achieve weight management during pregnancy especially from organisational factors that resulted in inadequate provider interactions and stigmatisation. Additionally at the policy level an over emphasis on providing information about the risks and a lack of gestational weight management guidance was also detrimental to women’s successful weight management.

The multi-factorial barriers identified within this research highlight the complexity of weight management in pregnant women with a raised BMI. While previous research has mainly concentrated on the woman as an individual, the need for a wider systems approach for effective management of obesity during pregnancy has been shown [65]. This requires a focus not just on the woman but also on wider organisational, community and policy factors that can impact on a women’s ability to achieve weight management [65]. Moving forwards, to better address the complexity, a better understanding of women’s motivation, capability or opportunity to change their behaviours during pregnancy is also required [66]. This necessitates a recognition of the competing demands on a woman’s attention during pregnancy, including financial, emotional and other health promotional activities [67]. Furthermore, women need support to implement the advice given [28,43] rather than assuming that education alone leads to behaviour change [68], as the majority of women with overweight or obesity indicate that they would like to make diet or physical activity related behaviour changes during pregnancy but only half feel confident to do so [69]. Providing women with a better understanding of the psychological and social context of their eating is essential to achieve this [43]. Furthermore, the amount of control each woman feels she has over her weight requires exploration, alongside her motivation to change [51] so that tailored support can be provided to women on how to implement advice that is given.

### Strengths and limitations

This study exclusively recruited women with a BMI≥40kg/m², whose voices are frequently missing within the literature around weight management during pregnancy. Several limitations were however acknowledged including that the women were only recruited from one setting and that while the sample was representative of the local population, with all women being from a White background, the views of women from other ethnicities were therefore not provided. Furthermore, six women who initially expressed an interest could not be followed up, although this is not too surprising given the proximity of all women to the due date of their baby when approached. Finally, the women who consented may have held stronger views on weight management services during pregnancy than the population in general.

It was ensured within the research that the interviewers were not involved in the women’s care in any way. As part of the process of reflexivity, which is essential in qualitative research since no research occurs within a vacuum [32,33] it was noted that the researcher who undertook the face-to-face interviews was a health professional with a BMI in the normal range. This may have influenced what the women themselves felt comfortable with sharing during the interviews.

### Implications for policy and practice and research

Weight management services during pregnancy need to be sensitive, respectful and centred on the individual woman and her current and previous experiences to reduce the stigmatisation that many women currently feel. To empower women, services should particularly focus on the provision of practical information, however this should not simply become a ‘tick box’ exercise. Information provision should follow a personalised approach that identifies and adapts to each individual woman’s information needs. To effectively achieve this and to enhance professional’s confidence around addressing weight management, all professionals require training on addressing the complex psychological and social context of weight for each woman, not just on the additional risks that women with obesity face during pregnancy. Furthermore, incorporating a range of behaviour change techniques when developing weight management services and focussing not just on individual factors, but other socio-ecological factors such as organisational, community and policy aspects is recommended to cater for women at different stages and with different barriers to weight management [51]. Given the higher weight gain among primigravid women with a BMI≥40kg/m², it could be argued that there is an additional requirement to focus on their needs. Services should also give more consideration to the advice and support women receive to tackle their weight in the postpartum period. Changes to current UK guidance to balance the focus on risk against practical recommendations is also required to combat stigma within maternity care. This requires further robust evidence of what interventions are effective at promoting weight management during pregnancy, particularly among women with obesity and within the UK context. A move away from exclusively using a medical, risk focussed, model of care is required to attend more satisfactorily to women’s individual needs.

## Conclusions

Women with obesity brought into pregnancy their history of past efforts at weight management which impacted upon their current motivation to engage with weight management advice. Services therefore need to be centred on individual women’s needs. Professionals faced a challenging task to ensure that women received adequate information without leaving them feeling stigmatised. Advising women of the risks associated with obesity during pregnancy left women feeling disempowered, unless there was concurrent provision of clear and consistent advice regarding healthy lifestyles and appropriate gestational weight gain.

## Supporting information

S1 File Interview schedule. (DOCX)
