# Supplementary material for: “Everything is revolved around me being heavy … it’s always, always spoken about.” Qualitative experiences of weight management during pregnancy in women with a BMI of 40kg/m2 or above
Source: PLoS One. 2022 Jun 24;17(6):e0270470. doi: 10.1371/journal.pone.0270470 (PMC9231696; doi:10.1371/journal.pone.0270470)
Supplement: S1 File — (DOCX) [file pone.0270470.s001.docx]

# Supplementary Information File 1: Interview schedule

Today I want to concentrate on how you feel about the maternal healthy weight service you received. There are no right or wrong opinions; I would like you to feel comfortable saying what you really think and how you really feel.

Has anyone discussed weight management with you during your pregnancy?

- - who has discussed it?
  - what has been discussed?
  - were they supportive in the way they discussed it?
  - was the information you were given useful/ or helpful?
  - was the information you got from different people consistent?
  - did you follow any of the advice you were given during pregnancy?
    - if yes - what?
    - how?
    - what did you find easy to do?
    - what was difficult to do?
    - if no - why not?
    - what did you find particularly difficult?
    - could anything have been done to make it easier?

*(prompt with any further discussions around:*

- *appropriate gestational weight gain*
- *healthy eating*
- *physical activity)*
- Have you been weighed at any point in pregnancy?
  - how did you feel about being weighed during pregnancy?
  - how often were you weighed?
  - and by who?
- Have you accessed any community based weight management services during pregnancy? ( eg exercise groups, Slimming world. weight watchers etc)
- Are you aware of any that you could access but didn't?

For anyone who has had a previous pregnancy:

- How did service provision in this pregnancy differ from service provision in previous pregnancies?

*If previous weight management service is mentioned:*

- *What were your initial thoughts about the service and being referred to it?*
- *Did you attend the service?*
- *Could you tell me about any parts you particularly liked?*
  - *why did you like them?*
  - *did you feel it was beneficial and helped you in any way?*
  - *could you share an example?*
- *Were there any aspects you didn't like?*
  - *why not?*
  - *what specifically didn't you like about it?*

So thinking of service provision in the future:

- Who would you feel most comfortable getting advice on weight management from?
- What, if anything, do you think could be improved in the services that you have received?
- What barriers do you think women face if following weight management advice during pregnancy?
  - Is there any way health professionals could help women to overcome these?
- What things do you think help following weight management advice during pregnancy?
  - Is there any way health professionals could help women to take advantage of these?
- Do you have any further comments you would like to make about the services provided or how best to support pregnant women to live healthy lifestyles?

Thank you again for taking part, we really appreciate it.
